# The Finite Element Analysis for a Mini-Conductance Probe in Horizontal Oil-Water Two-Phase Flow

**DOI:** 10.3390/s16091352

**Published:** 2016-08-24

**Authors:** Weihang Kong, Lingfu Kong, Lei Li, Xingbin Liu, Ronghua Xie, Jun Li, Haitao Tang

**Affiliations:** 1 School of Information Science and Engineering, Yanshan University, Qinhuangdao 066004, Hebei, China; kongweihang@163.com (W.K.); dlts_lil@petrochina.com.cn (L.L.); 2 Key Laboratory for Computer Virtual Technology and System Integration of Hebei Province, Qinhuangdao 066004, Hebei, China; 3 Logging and Testing Services Company, Daqing Oilfield Limited Company, Daqing 163453, Heilongjiang, China; dlts_liuxb@petrochina.com.cn (X.L.); dlts_lijun@petrochina.com.cn (J.L.); dlts_tanght@petrochina.com.cn (H.T.); 4 Daqing Oilfield Limited Company, Daqing 163453, Heilongjiang, China; xieronghua@petrochina.com.cn

**Keywords:** horizontal oil-water segregated flow, mini-conductance probe (MCP), finite element method (FEM), sensitivity field, design and geometry optimization, pure-water phase conductivity measurement

## Abstract

Oil-water two-phase flow is widespread in petroleum industry processes. The study of oil-water two-phase flow in horizontal pipes and the liquid holdup measurement of oil-water two-phase flow are of great importance for the optimization of the oil production process. This paper presents a novel sensor, i.e., a mini-conductance probe (MCP) for measuring pure-water phase conductivity of oil-water segregated flow in horizontal pipes. The MCP solves the difficult problem of obtaining the pure-water correction for water holdup measurements by using a ring-shaped conductivity water-cut meter (RSCWCM). Firstly, using the finite element method (FEM), the spatial sensitivity field of the MCP is investigated and the optimized MCP geometry structure is determined in terms of the characteristic parameters. Then, the responses of the MCP for the oil-water segregated flow are calculated, and it is found that the MCP has better stability and sensitivity to the variation of water-layer thickness in the condition of high water holdup and low flow velocity. Finally, the static experiments for the oil-water segregated flow were carried out and a novel calibration method for pure-water phase conductivity measurements was presented. The validity of the pure-water phase conductivity measurement with segregated flow in horizontal pipes was verified by experimental results.

## 1. Introduction

Two-phase flow is widespread in the petroleum industry, chemical engineering, pharmacy and nuclear reaction fields, etc. The study of liquid-liquid/gas-liquid two-phase flow in horizontal pipes and the individual phase concentration measurement of liquid-liquid/gas-liquid two-phase flow are of great importance for diverse industrial applications and the optimization of the oil production process [1,2]. Electrical methods (the capacitance method and conductance method) are widely used for gas-liquid/liquid-liquid two-phase flow measurements because they are characterized by their clarity of principle, simple structure, easy implementation, low cost, on-line measurement, fast response, and lack of fluid interference, etc. [3,4,5,6,7,8,9]. In particular the conductance method [8,9] is mainly dependent on the conductive properties of the flow components, which means that a direct measurement of phase holdup could be made if the two flow components are both electrically conductive and have contrasting conductivity. Therefore, it is very important and meaningful to measure the water holdup of oil-water two-phase flow in horizontal wells by using the conductance method [10].

The measurement principle of water holdup [1,11,12] indicates that water holdup can be determined by oil-water mixture phase conductivity and continuous water phase conductivity. Water holdup, which can be obtained according to Maxwell analytic model [13], can be converted into a water volume fraction, with an interpretation chart for slip correction. The value of oil-water mixture phase conductivity is mainly dependent on the dispersed oil-phase volume fraction and continuous water phase conductivity; meanwhile it is influenced by the space distribution of the discrete phase.

In terms of the ring-shaped conductance probe (RSCP) geometry optimization, Lucas et al. [14,15] used the FEM to investigate the sensitivity field distribution for the geometry optimization of electrodes. Fossa and Devia [16,17] optimized the RSCP geometry, and investigated the effects of the RSCP geometry on the response of the measuring device in annular, segregated and bubble flows, and improved the RSCP response both in terms of linearity and spatial resolution by means of the numerical solution of Laplace problem. Using the FEM, Jin et al. [18] designed and optimized a conductivity probe with a vertical multiple electrode array (VMEA), proposed the content of the spatial sensitivity and effective information, and then, constructed an optimized VMEA which could be used to measure cross-correlation velocity and predict volume fraction in vertical upward gas-water two-phase flow with satisfactory accuracy.

Although progress has been made on the conductance method in previous research, the early work mainly focused on the investigation of the sensitivity distribution of the electric field for vertical small diameter pipes, and paid little attention to the effects of various flow patterns in horizontal pipes on the output response both in terms of linearity and spatial resolution. The oil-water two-phase flow patterns of long distance undulating horizontal wells have a mixed flow pattern which is mainly horizontal segregated flow as opposed to vertical wells [2,19]. The flow pattern and flow velocity are influenced by the inclination of the long distance undulating horizontal well, so the pre-existing profile parameters measuring sensor production for vertical wells cannot be directly used for horizontal wells.

Early experimental investigations on the horizontal oil-water two-phase flow patterns were conducted in acrylic pipes with a small diameter, and the flow patterns were primarily defined by simple observations [20,21]. Arirachakaran et al. [22] observed stratified flow, mixed flow, annular flow, intermittent flow and dispersed flow in a 25.1 mm inner-diameter (ID) horizontal pipe. Trallero et al. [23] comprehensively performed an experimental and theoretical study in a 50.1 mm ID pipe, and then classified the flow patterns into segregated flow and dispersed flow, the segregated flow includes stratified flow (ST) and stratified flow with mixing at the interface (ST & MI), the dispersed flow includes dispersion of oil in water and water flow (DO/W & W), dispersion of water in oil and oil in water flow (DW/O & DO/W), dispersion of oil in water flow (DO/W), and dispersion of water in oil flow (DW/O). Zhai et al. [24] conducted an experimental under various flow conditions in a 20 mm ID horizontal pipe by using the radial mini-conductance probes and RSCP, analyzed and obtained the same result of flow patterns classification*.*

In vertical production profile logging wells, the RSCWCM measures oil-water mixture phase conductivity online when the fluid flows through the RSCP measuring pipelines. The pure-water phase conductivity could be obtained by the RSCP connected with a sampler for oil-water two-phase separation [1,12]. In horizontal production profile logging wells, the oil-water mixture phase conductivity could be measured online in the process of fluid flow by using the RSCWCM [25]. However, due to the characteristics of the horizontal well structure and limitations of the ring-shaped electrode structure of the RSCWCM, the RSCP must still be immersed in the oil-water segregated flow when sampling [26], so it is impossible to realize pure-water result sampling calibration, and the RSCWCM cannot be applied in horizontal wells.

In this study, we designed and optimized a MCP for measuring pure-water phase conductivity of oil-water two-phase segregated flows in horizontal pipes. We also present the experimental results to verify the validity of pure-water phase conductivity measurement using our new MCP.

## 2. Simulation of the MCP

### 2.1. The Geometry of the MCP

**Figure 1:**
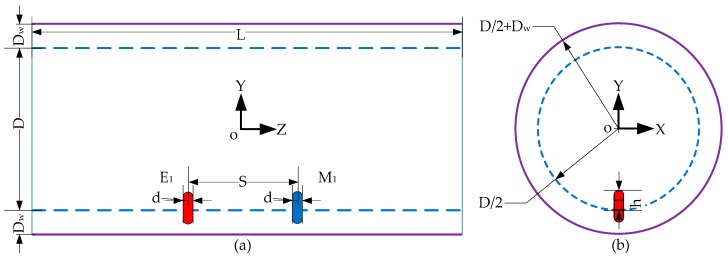
The geometric structure and the parameters of the MCP: (**a**) the axial section; (**b**) the cross section.

Figure 1 shows the geometric structure and the parameters of the MCP. The MCP consists of a pair of metal parallel-electrodes along the axial section direction, with each electrode flush-mounted on the inside wall of the insulated flow pipe. E_1_ and M_1_ represent two electrodes, each of which is simultaneously the exciting and measuring electrode. Polarization phenomena occur in the vicinity of the E_1_ and M_1_ electrodes when the MCP is excited by low frequency electrical power, which would result in parasitic impedance and lead to a detectable measuring error.

The structure parameters and coordinates of the MCP are also shown in Figure 1, in which *S* is the separation between the two electrodes, *d* is the same diameter of metal parallel-electrodes, *h* is the measurement field height of the metal parallel-electrode, *D* is the inner diameter of the insulated flow pipe, *D*_w_ is the thickness of the insulated flow pipe, and *L* is the axial length of the insulated flow pipe.

### 2.2. The Meshed Model of the MCP

Since the measurement field of the MCP is much smaller than the wavelength of the electric field, the electric field can be modeled as time invariant. With this regard, it uses a direct current source is used as the exciting signal to investigate the electric field of the MCP. The distribution of the electric field can be described by the Laplace equation [27]:

(1) $$\nabla^{2}u(r,\varphi ,z)=\frac{1}{r}\frac{\partial }{\partial r}\left(r\frac{\partial u}{\partial r}\right)+\frac{1}{r^{2}}\frac{\partial^{2}u}{\partial \varphi }+\frac{\partial^{2}u}{\partial z^{2}}=0$$

The essential boundary conditions can be expressed as

(2) $$\left\{ \begin{array}{l} u=0\;(r\in [\frac{D}{2}-h,\frac{D}{2}],\varphi \in [-arctan(\frac{d}{D-2h}),+arctan(\frac{d}{D-2h})],z\in [\frac{S-d}{2},\frac{S+d}{2}])\\ \frac{\partial u}{\partial r}=\frac{I_{e}}{S_{d}}\;(r\in [\frac{D}{2}-h,\frac{D}{2}],\varphi \in [-arctan(\frac{d}{D-2h}),+arctan(\frac{d}{D-2h})],z\in [-\frac{S+d}{2},-\frac{S-d}{2}])\\ \frac{\partial u}{\partial z}=0\;(z=\pm \frac{L}{2}) \end{array}\right.$$

where *u* is the inner electric potential distribution, *I*_e_ represents exciting current, *D* is the inner diameter of the insulated flow pipe, *L* is the insulated flow pipe length, *h* is the measurement field height of the metal parallel-electrode, and *S_d_* is the surface area of the two electrodes of the MCP.

**Figure 2:**
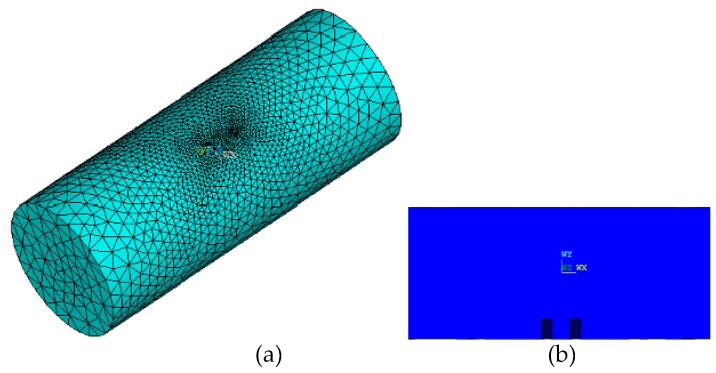
The meshed 3D model of the MCP with 26,695 elements, *L* = 0.05 m, *D* = 0.02 m, *S* = 0.004 m, *d* = 0.0015 m, *h* = 0.002 m, water resistivity σ_w_ = 100 Ω·m: (**a**) 3D model; (**b**) the axial section.

We employ the FEM to investigate the distribution of the electric field inside the MCP. Figure 2 shows the meshed finite element model of the MCP in ANSYS software in which we chose SOLID232 as the element type and used free meshing to mesh the model. The total element number of the units of the meshed finite model is 26,695. A direct current of 0.1 mA is supplied to the electrode E_1_, a current of −0.1 mA is supplied to M_1_, and the voltage of electrode M_1_ is simultaneously set to 0 V.

### 2.3. Optimize Parameters for the MCP Structure

The relative change amount of difference of output voltage between the two electrodes are measured after putting an oil droplet at some sensitive position coordinates (*x*,*y*,*z*), where the measuring electrodes difference of output voltage U_0_ is used as a reference when the insulated flow pipe is only filled with a continuous water phase. Using the finite element simulation model of the MCP it could be known that when the oil droplet is located at any unit volume in the measurement field, the response voltage of the measuring electrodes is U*_m_*(*x*,*y*,*z*) and the difference of output voltage between the measuring electrodes is ΔU*_m_*(*x*,*y*,*z*). The element sensitivity $\psi_{m}(x,y,z)$ is the result of the normalization for the relative change amount of difference of output voltage at any unit volume in the spatial measurement field, which is generated by the conductivity change of the subdomain. The element sensitivity $\psi_{m}(x,y,z)$ can be expressed as [14]:

(3) $$\Delta U_{m}(x,y,z)=U_{m}\left(x,y,z\right){\text{-U}}_{0}\;m=1,2,\cdots ,N$$

(4) $$\psi_{m}(x,y,z)=\frac{\Delta U_{m}(x,y,z)}{{\left[\Delta U_{m}(x,y,z)\right]}_{\text{max}}}\times 100\%\;m=1,2,\cdots ,N$$

where [ΔU*_m_*(*x*,*y*,*z*)]_max_ is the maximum change in the value of the voltage drop between electrodes E_1_ and M_1_ that was calculated for the range of particle positions under consideration, and *N* is the total number of the unit volume in the measurement field.

The spatial sensitivity is the set of the sensitivity for any element unit volume in the spatial measurement field. In the process of simulation, using the insulated oil droplet as the test particle to traverse for the results of the equal division quantization for the volume of the spatial measurement field, from which the change amount of voltage at any element unit volume can be obtained. The element sensitivity can then be obtained after the normalization for the change amount of voltage at any element unit volume, which has nothing to do with the volume of the oil droplet.

The test particle was regarded as the center of mass to process while obtaining the element sensitivity in the spatial measurement field, that is, traversing the spatial measurement field is implemented by changing the center of mass position of the test particle.

This paper obtained the set of the sensitivity for any element unit volume of any center of mass by changing the center of mass with some regularity during obtaining the average spatial sensitivity in the spatial measurement filed of sensors, that is, the space measurement field is quantified into *N* equal parts, and *S_avg_* can be accessed by averaging the *N* parts of each elementsensitivity, based on the investigation into the sensor sensitivity distribution [28].

Taking into account the computational speed of the FEM in the three-dimensional electric field, an oil droplet of 2 mm-diameter was used in the simulation experiment. Changing the center of mass for the oil droplet, and solving finite element model with formula (1), the element sensitivity distribution of the MCP in the specific structure can then be obtained:

(5) $$S_{avg}=\frac{1}{N}\sum_{m=1}^{N}\psi_{m}(x,y,z)\;\;m=1,2,\cdots ,N$$

The sensitivity variation parameter *S_vp_*:

(6) $$S_{vp}=\frac{S_{dev}}{S_{avg}}\times 100\%$$

The parameters *S_avg_* and *S_vp_* are used as the optimization indexes to study the effect of sensor geometry on the sensitivity field, in which *S_vp_* is the standard deviation of the element sensitivity and can be expressed as:

(7) $$S_{dev}={\left[\frac{1}{N}\sum_{m=1}^{N}{\left(\psi_{m}(x,y,z)-S_{avg}\right)}^{2}\right]}^{1/2}\;\;m=1,2,\cdots ,N$$

Obviously, the smaller the parameter *S_vp_* is, the more homogeneous the distribution of the sensor sensitivity field would be. This indicates that the sensitivity of the sensor is better when *S_avg_* is larger. When *S_vp_* is smaller, the detection sensitivity is more uniformand the measurement value error is smaller. Actually, we expected to get high sensor relative sensitivity *S_avg_* and *S_vp_* minimized at the same time when designing and optimizing the MCP.

In the process of sensor parameters optimization, the electric field distribution evenness and sensor sensitivity distribution uniformity are used as the performance indicators of the MCP. The parameters *d*, *h* and *S* are the main factors that influence the MCP spatial sensitivity distribution. Especially the parameter *h* directly determines the ability of the MCP to distinguish the level of oil-water contact interface for pure-water phase conductivity measurements. Therefore, in this paper, under the condition of the parameter *D* = 20 mm, the parameter *h* is set to 3, 2 and 1 mm, respectively, to analyze the electric field distribution and sensitivity intensity distribution of the MCP in simulation and parameter optimization.

#### 2.3.1. Electric Field Distribution and Parameters Optimization When *h* = 3 mm

**Figure 3:**
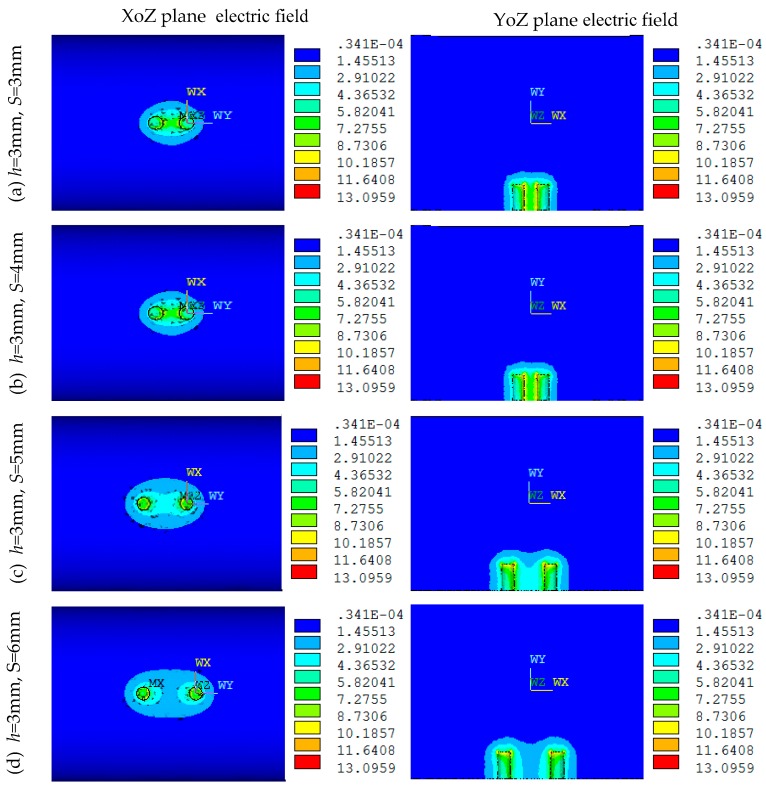
Electric field distribution of the MCP under different *S* values (*D* = 20 mm, *h* = 3 mm, *d* = 1.5 mm): (**a**) *S* = 3 mm; (**b**) *S* = 4 mm; (**c**) *S* = 5 mm; (**d**) *S* = 6 mm.

Figure 3 shows the electric field distributions of both the XoZ and YoZ sections of the MCP in simulation when *D* = 20 mm, *h* = 3 mm, *d* = 1.5 mm, *S* equals 3, 4, 5, 6 mm, respectively. As can be seen in Figure 3, the *S* has great influences on the electric field distribution of the MCP. The larger the *S* is, the weaker the electric field between the two electrodes is, and the more uneven the distribution is. When *S* = 3 mm, the electric field intensity between the two electrodes is large, but the distribution uniformity is influenced; when *S* = 6 mm, the electric field intensity between the two electrodes is significantly decreased, the distribution uniformity is severely influenced.

Based on the study of the sensor electric field using FEM, we can further calculate the 2D sensitivity distribution between two measuring electrodes of the MCP. In the calculation, the relative resistivity of water and oil are respectively set as 100 Ω·m and 1.0*E* + 13 Ω·m. It is worth nothing that the center of mass of the oil droplet is located on the YoZ plane section. The total number of the test positions *N* is equal to 399.

**Figure 4:**
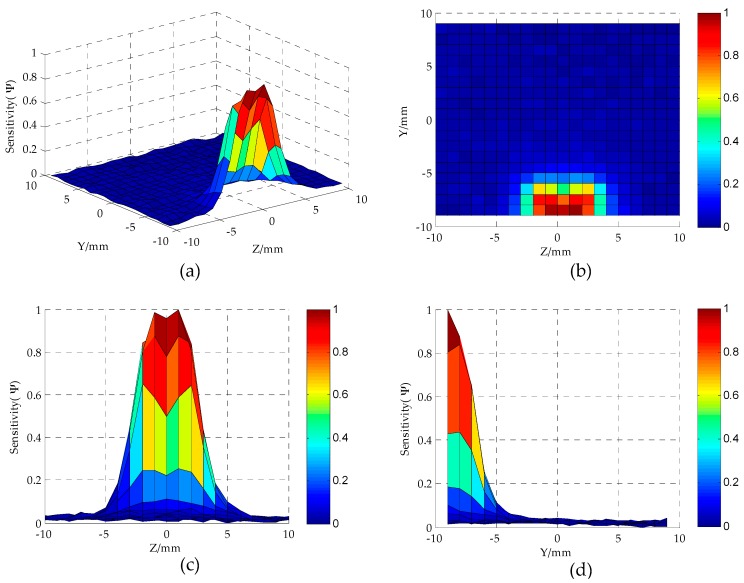
Sensor sensitivity distribution in YoZ plane sensitive field (*D* = 20 mm, *h* = 3 mm, *d* = 1.5 mm, *S* = 4 mm): (**a**,**b**) YoZ plane regions; (**c**) *Z*-axis direction; (**d**) *Y*-axis direction.

As can be seen from Figure 3, when *S* = 4 mm, the electric field distribution of the MCP is relatively uniform. Figure 4 shows the distribution of sensor sensitivity field with the parameters constant at *D* = 20 mm, *h* = 3 mm, *d* = 1.5 mm and *S* = 4 mm. As can be seen from Figure 4a,b, the regions around the electrodes show relatively high sensitivity, while away from the electrodes the sensitivity tends to be quite low. As can be seen from Figure 4b–d, the regions show relatively high sensitivity at the position of |Z| < 6 mm and Y < −4 mm; the sensor is not sensible to oil droplet in the region of |Z| > 6 mm or Y > −4 mm.

The sensor relative sensitivity *S_avg_* and sensitivity variation parameter *S_vp_* extracted from Figure 4 are 0.104 and 1.779, respectively. Furthermore, the dependence of the calculated sensitivity field on the number of the meshed grids in the finite model is investigated. As shown in Table 1, it is obvious that the value of *S_avg_*, as well as the value of *S_vp_*, has a very small differences in different grids, which indicates the relative independence of the calculated results on the meshed grids.

**Figure 5:**
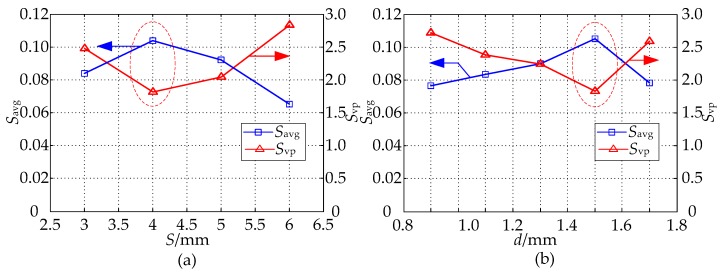
The effect of the MCP geometry on *S_avg_* and *S_vp_*: (**a**) effect of the parameter *S* (*D* = 20 mm, *h* = 3 mm, *d* = 1.5 mm); (**b**) effect of the parameter *d* (*D* = 20 mm, *h* = 3 mm, *S* = 4 mm).

Under the condition of the parameter *D* = 20 mm, to further investigate the influence which the parameters *S* and *d* have on sensitivity distribution when *h* = 3 mm, in simulation calculation, by obtaining the sensitivity values of different sensor geometries in the sensitive field, we use the sensitivity optimization indexes to optimize the parameters *S* and *d*, and then get their optimal values. The range of parameters *S* and *d* are (3 mm, 6 mm), (0.9 mm, 1.7 mm), respectively. Figure 5 shows the optimization indexes *S_avg_* and *S_vp_* of the MCP sensitivity field in which both the *S* and *d* are different. In Figure 5a by setting *h* = 3 mm, *d* = 1.5 mm, the *S_avg_* and *S_vp_* of the MCP sensitivity field in which *S* is different are calculated. From the analysis of Figure 3 and Figure 5a, when *S* = 4 mm, the relative sensitivity in sensitive field has a higher value, and its distribution is relatively uniform. In Figure 5b, on the basis of the optimized parameter *S*, with the parameters constant at *h* = 3 mm and *S* = 4 mm, and the *S_avg_* and *S_vp_* of the MCP sensitivity field in which *d* is different are calculated. As can be seen in Figure 5b, the relative sensitivity has a higher value in sensitive field, and its distribution is relatively uniform when *d* = 1.5 mm.

According to the characteristic parameters of the sensor sensitivity field, when the parameters *D* = 20 mm and *h* = 3 mm, we select the optimum geometry of MCP as *S* = 4 mm, *d* = 1.5 mm.

#### 2.3.2. Electric Field Distribution and Parameters Optimization When *h* = 2 mm

**Figure 6:**
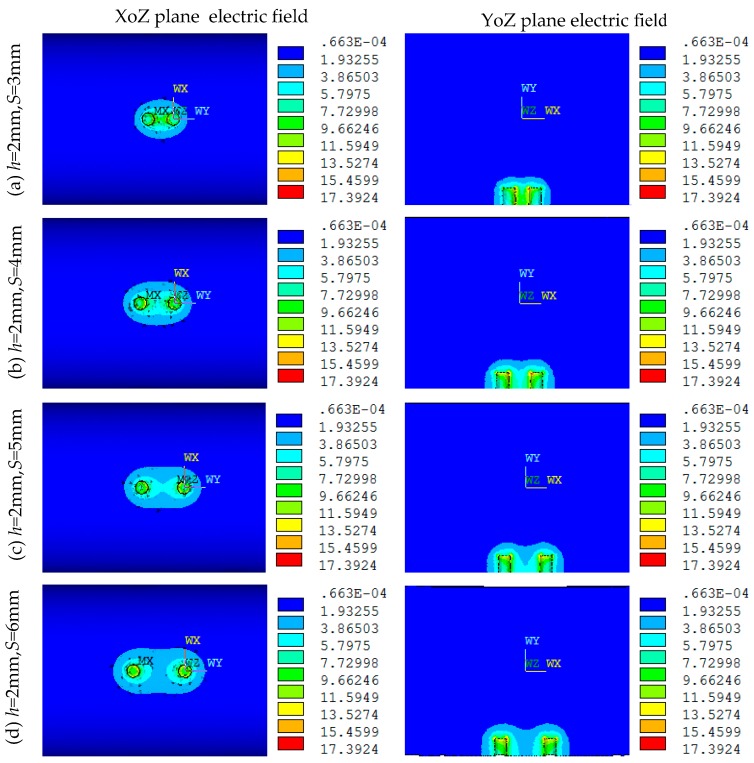
Electric field distribution under different *S* (*D* = 20 mm, *h* = 2 mm, *d* = 1.5 mm): (**a**) *S* = 3 mm; (**b**) *S* = 4 mm; (**c**) *S* = 5 mm; (**d**) *S* = 6 mm.

Figure 6 shows the electric field distributions of both the XoZ and YoZ sections of the MCP when the parameters *D* = 20 mm, *h* = 2 mm, *d* = 1.5 mm, and *S* equals to 3, 4, 5, 6 mm, respectively. As can be seenin Figure 6, *S* has a great influence on the electric field distribution of the MCP, and the greater the *S* is, the weaker and the more unevenly distributed the electric field is. When *S* = 3 mm, the intensity of the electric field between the two electrodes is larger, but the distribution uniformity is affected. When *S* = 6 mm, the electric field intensity between the two electrodes decreases significantly, and distribution uniformity is severely affected.

**Figure 7:**
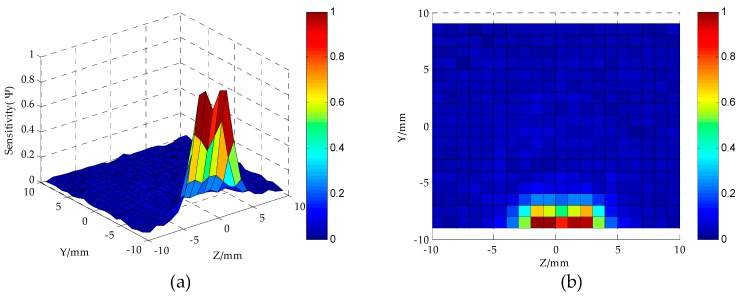

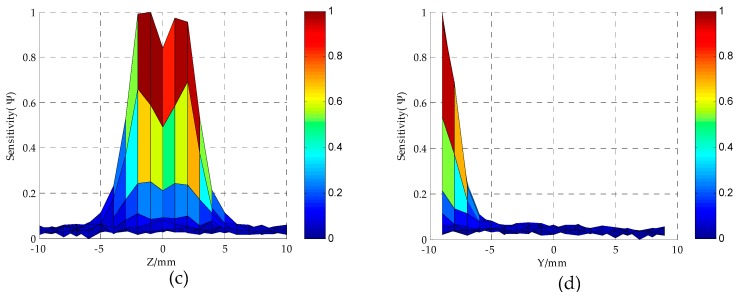
Sensor sensitivity distribution in YoZ plane sensitive field (*D* = 20 mm, *h* = 2 mm, *d* = 1.5 mm, *S* = 4 mm): (**a**,**b**) YoZ plane regions; (**c**) *Z*-axis direction; (**d**) *Y*-axis direction.

As can be seen from Figure 6, when *S* = 4 mm, the electric field distribution of the MCP is relatively uniform. Figure 7 shows the distribution of sensor sensitivity field with the parameters constant at *D* = 20 mm, *h* = 2 mm, *d* = 1.5 mm and *S* = 4 mm. As can be seen from Figure 7a,b, the regions around the electrodes show relatively high sensitivity, while away from the electrodes the sensitivity tends to be quite low. As can be seen from Figure 7b–d, the regions show relatively high sensitivity at the position of |Z| < 6 mm and Y < −5 mm; the sensor is not sensitive to oil droplets in the region of |Z| > 6 mm or Y > −5 mm.

**Figure 8:**
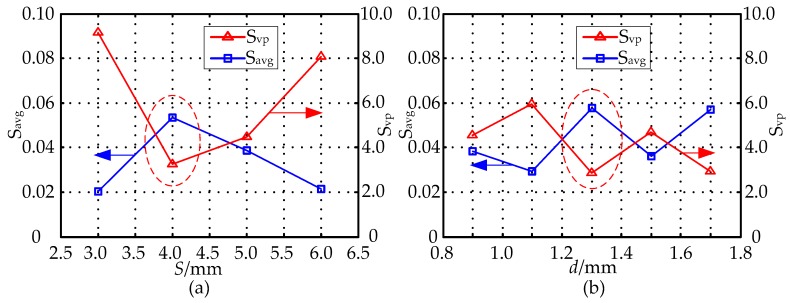
The effect of the MCP geometry on *S_avg_* and *S_vp_*: (**a**) effect of the parameter *S* (*D* = 20 mm, *h* = 2 mm, *d* = 1.5 mm); (**b**) effect of the parameter *d* (*D* = 20 mm, *h* = 2 mm, *S* = 4 mm).

Under the condition of the parameter *D* = 20 mm, to further investigate the influences the parameters *S* and *d* have on the sensitivity distribution when *h* = 2 mm, in simulation experiment, by obtaining the sensitivity values of different sensor geometriesin the sensitive field, we use the sensitivity optimization indexes to optimize the parameters *S* and *d*, and then get their optimal values. The range of parameters *S*, *d* are (3 mm, 6 mm), (0.9 mm, 1.7 mm), respectively. Figure 8 shows the optimization indexes *S_avg_* and *S_vp_* of MCP sensitivity field in which both the *S* and *d* are different. In Figure 8a by setting *D* = 20 mm, *h* = 2 mm, *d* = 1.5 mm, respectively, the optimization indexes *S_avg_* and *S_vp_* of MCP sensitivity field in which *S* is different are calculated. From the analysis of Figure 7 and Figure 8a, when *S* = 4 mm, the relative sensitivity has a higher value in sensitive field, and its distribution is relatively uniform. In Figure 8b, on the basis of the optimized parameter *S*, with the parameters constant at *D* = 20 mm*, h* = 2 mm and *S* = 4 mm, the optimization indexes *S_avg_* and *S_vp_* of the MCP sensitivity field in which *d* is different are calculated. Figure 8b shows that the relative sensitivity has a higher value in sensitive field, and its distribution is relatively uniform when *d* = 1.3 mm. According to the characteristic parameters of the sensor sensitivity field, when *D* = 20 mm, *h* = 2 mm, the optimum geometry of MCP is selected as *S* = 4 mm, *d* = 1.3 mm.

#### 2.3.3. Electric Field Distribution and Parameters Optimization When *h* = 1 mm

**Figure 9:**
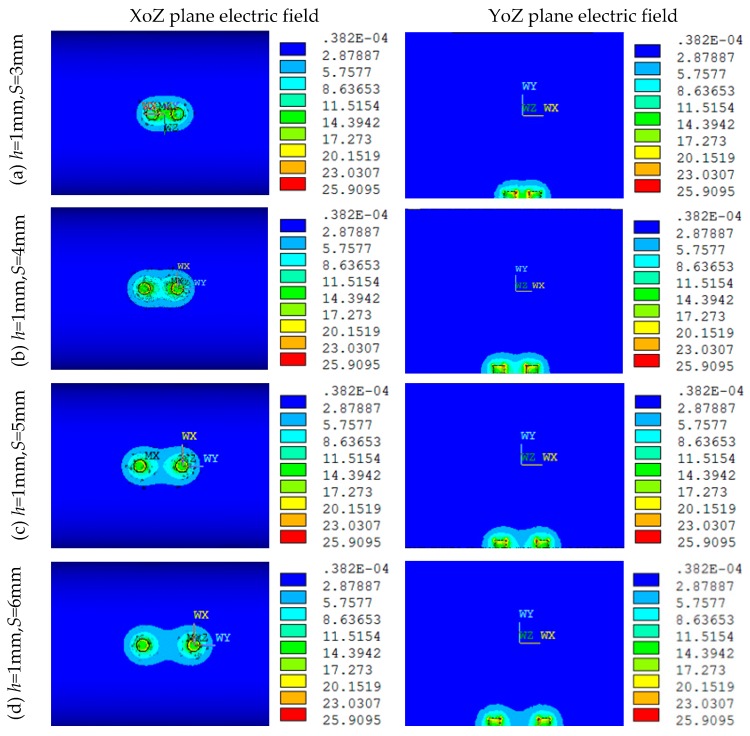
Electric field distribution under different *S* (*D* = 20 mm, *h* = 1 mm, *d* = 1.5 mm): (**a**) *S* = 3 mm; (**b**) *S* = 4 mm; (**c**) *S* = 5 mm; (**d**) *S* = 6 mm.

Figure 9 shows the electric field distributions of both the XoZ and YoZ sections of the MCP when *D* = 20 mm, *h* = 1 mm, *d* = 1.5 mm, and *S* equals to 3, 4, 5, 6 mm, respectively. As can be observed from Figure 9, the parameter *S* has a great influence on the electric field distribution of the MCP, and the greater the *S* is, the weaker the electric field is, and the more uneven the distribution is. When *S* = 3 mm and *S* = 4 mm, the intensity of the electric field between the two electrodes is larger, and the distribution uniformity is affected. When *S* = 5 mm and *S* = 6 mm, the electric field intensity between the two electrodes intensity significantly decreases, and the distribution uniformity is severely affected.

**Figure 10:**
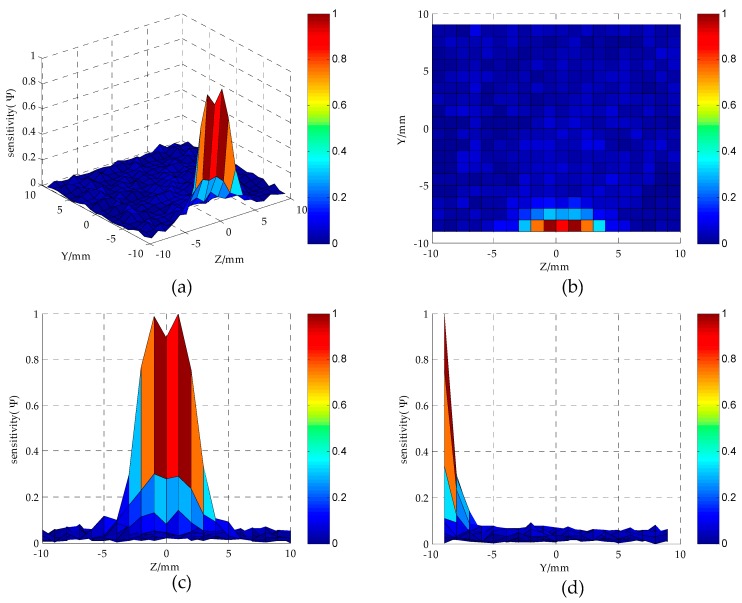
Sensor sensitivity distribution in YoZ plane sensitivity field (*D* = 20 mm, *h* = 1 mm, *d* = 1.5 mm, *S* = 3 mm): (**a**,**b**) YoZ plane regions; (**c**) *Z*-axis direction; (**d**) *Y*-axis direction.

As can be seen from Figure 9, when *S* = 3 mm, the electric field distribution of the MCP is relatively uniform. Figure 10 shows the distribution of sensor sensitivity field with the parameters constant at *D* = 20 mm, *h* = 1 mm, *d* = 1.5 mm and *S* = 3 mm. As can be seen from Figure 10a,b, the regions around the electrodes show relatively high sensitivity, while away from the electrodes the sensitivity tends to be quite low. As can be seen from Figure 10b–d, the regions show relatively high sensitivity at the position of |Z| < 5 mm and Y < −6 mm; the sensor is not sensible to oil droplet in the region of |Z| > 5 mm or Y > −6 mm.

**Figure 11:**
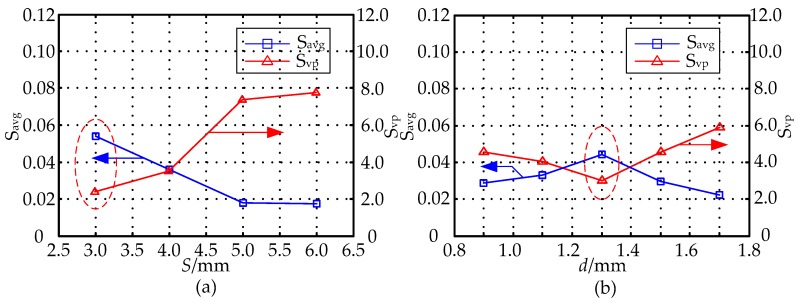
The effect of the MCP geometry on *S_avg_* and *S_vp_*: (**a**) effect of the *S* (*D* = 20 mm, *h* = 1 mm, *d* = 1.5 mm); (**b**) effect of the *d* (*D* = 20 mm, *h* = 1 mm, *S* = 3 mm).

To further investigate the influences of the parameters *S* and *d* have on sensitivity distribution when *D* = 20 mm, *h* = 1 mm, in simulation experiments, by obtaining the sensitivity values of different sensor geometries in the sensitive field, we use the sensitivity optimization indexes to optimize the parameters *S* and *d*, and then gets their optimal values. The range of parameters *S*, *d* are (3 mm, 6 mm), (0.9 mm, 1.7 mm), respectively. Figure 11 shows the effect of the MCP geometry on the optimization indexes *S_avg_* and *S_vp_* of the MCP sensitivity field in which the parameters *S* and *d* are different. In Figure 11a by setting *D* = 20 mm, *h* = 1 mm, *d* = 1.5 mm, respectively, the optimization indexes *S_avg_* and *S_vp_* of the MCP sensitivity field in which *S* is different are calculated. From the analysis of Figure 10 and Figure 11a, when *S* = 3 mm, the relative sensitivity in the sensitive field has a higher value, and its distribution is relatively uniform. In Figure 11b, on the basis of the optimized parameter *S*, with the parameters constant at *D* = 20 mm, *h* = 1 mm, *S* = 3 mm, the *S_avg_* and *S_vp_* of the MCP sensitivity field in which *d* is different are calculated. As can be seen in Figure 11b, the relative sensitivity has a higher value in sensitive field, and its distribution is relatively uniform when *d* = 1.3 mm.

According to the characteristic parameters of the sensor sensitivity field, when *D* = 20 mm, *h* = 1 mm, the optimum geometry of MCP is selected as *S* = 3 mm, *d* = 1.3 mm.

## 3. Simulation Calculated Response for Segregated Flow

From Section 2, the sensitive fields of the MCP with different values of *h* have some differences when the parameters *S* and *d* are constant, especially with the increase of the parameter *h*, the sensitive field of sensor along the *Y*-axis shows a continuous expanding tendency. Consequently, to measure the pure-water phase conductivity in the condition of the horizontal oil-water segregated flow, the effect degree of the MCP in the measurement field along the *Y*-axis by the non-conducting materials such as oil phase and so on needs further investigation. From the analysis of Figure 5, Figure 8 and Figure 11, under the condition of the parameter *D* = 20 mm, the geometry parameters *h*, *S* and *d* of the MCP after optimization value *h* = 3 mm, *S* = 4 mm, *d* = 1.5 mm and *h* = 2 mm, *S* = 4 mm, *d* = 1.3 mm and *h* = 1 mm, *S* = 3 mm, *d* = 1.3 mm, respectively.

**Figure 12:**
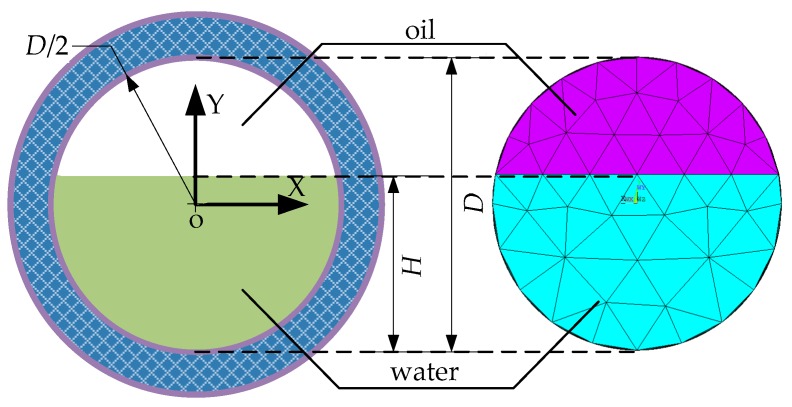
Oil-water contact interface and 2D view of the finite element model for segregated flow sketches.

In this simulation, the response of the MCP for oil-water segregated flow is calculated using a 3D finite element model. Figure 12 shows the 2D view of the meshed model for segregated flow. *H* is the thickness of the water layer, *D* is the inner diameter of the insulated flow pipe. The simulation conditions, which are also same as the condition in Section 2, are that the water resistivity *σ_w_* = 100 Ω·m, oil resistivity *σ_o_* = 1.0*E* + 13 Ω·m, a direct current of 0.1 mA is supplied to the electrode E_1_, a current of −0.1 mA is supplied to M_1_, and the voltage of electrode M_1_ is simultaneously set to 0 V.

**Figure 13:**
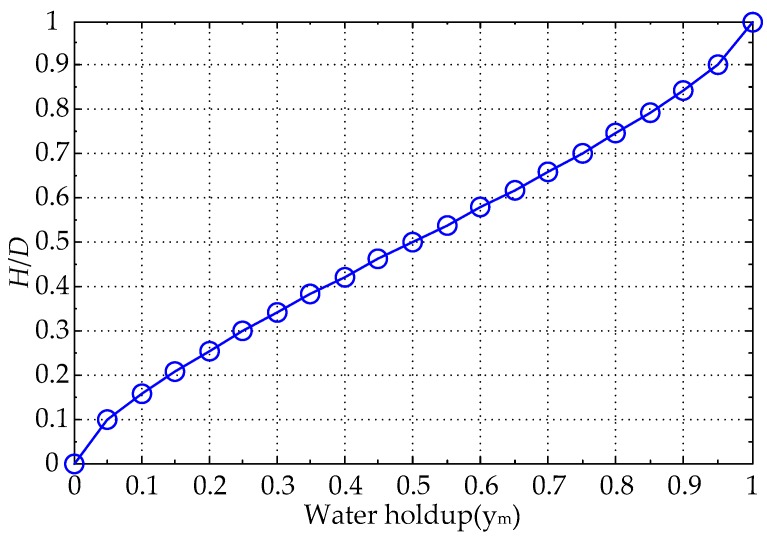
Water holdup *y_m_* and (*H*/*D*) curve.

Figure 13 shows the curve between water holdup *y_m_* and height of water layer (*H*/*D*), and it is a nonlinear curve between the *y_m_* and the (*H*/*D*), the range of both *y_m_* and (*H*/*D*) are from 0 to 1. When *y_m_* = 0 or (*H*/*D*) = 0, the insulated flow pipe is filled with pure oil; when *y_m_* = 1 or (*H*/*D*) = 1, the insulated flow pipe is filled with pure water; when 0 < *y_m_* < 1 or 0 < *H*/*D* < 1, the insulated flow pipe is filled with oil-water mixture.

**Figure 14:**
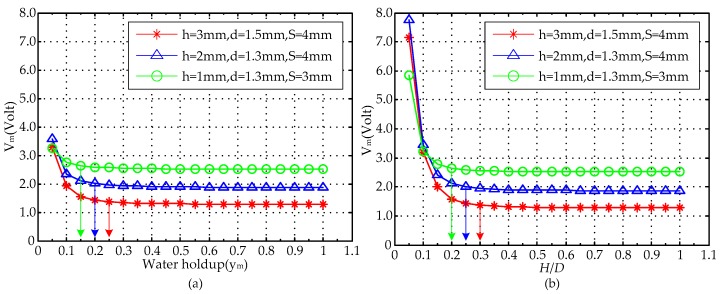
FEM calculated response of the MCP with *D* = 20 mm for segregated flow: (**a**) *V_m_* vs. *y_m_*; (**b**) *V_m_* vs. *H*/*D*.

Under the condition of the parameter *D* = 20 mm, the response signals of the MCP are analyzed in terms of *y_m_* and *H*/*D*, respectively, the range of both *y_m_* and *H*/*D* are from 0 to 1, and the change interval with 0.05 was used as an experimental point. The calculated response values of the MCP are shown in Table 2 and Figure 14 in which *V_m_* is the measured voltage and can be expressed as:

(8) $$V_{m}=k_{\sigma }\times \sigma_{m}\times I_{e}$$

where *k_σ_* is resistance correction coefficient, *σ_m_* is resistivity of oil-water mixture in measurement field, *I_e_* represents exciting current.

As can be seen from Table 2, the response values of the MCP have obvious differences under the pure oil and pure water conditions, which can be attributed to the distinctive difference of water and oil resistivity. Meanwhile, the response values of sensor structure with different parameters are different, which can be attributed to the different electric field generated by two electrodes in measurement field.

As can be seen from Figure 14, the range of both *y_m_* and *H*/*D* are from 0.05 to 1, and with the increase of *y_m_* or *H*/*D*, under the condition of lower *y_m_* or *H*/*D*, the measured voltage value *V_m_* shows an obvious difference and decreasing tendency, which can be attributed to the stronger electric field in the measurement region, where the non-conducting oil phase is located in the high sensitivity sensitive area analyzed from Figure 4, Figure 7 and Figure 10. Therefore, the response result is seriously affected by the non-conducting oil phase and the pure-water result cannot be obtained. When *y_m_* or *H*/*D* is greater than or equal to a constant value, the response result of the MCP is approximately equal to pure-water value, and it is almost not influenced by oil phase, which can be attributed to the non-conducting fluid away from the high sensitivity sensitive area. Under the conditions of higher *y_m_* or *H*/*D*, the MCP can obtain a reliable and accurate pure-water value, and it can be used as the pure-water correction for water content measurement. As can be seen from Figure 14a, in the condition of the MCP with *D* = 20 mm, *h* = 3 mm, *d* = 1.5 mm and *S* = 4 mm, the response result is approximately equal to the pure-water value when *y_m_* is greater than or equal to 0.25. In the condition of the MCP with *D* = 20 mm, *h* = 2 mm, *d* = 1.3 mm and *S* = 4 mm, the response result is approximately equal to the pure-water value when *y_m_* is greater than or equal to 0.20. In the condition that the MCP with *D* = 20 mm, *h* = 1 mm, *d* = 1.3 mm and *S* = 3 mm, the response result is approximately equal to the pure-water value when *y_m_* is greater than or equal to 0.15. As well, in Figure 14b, in the condition of the MCP with *D* = 20 mm, *h* = 3 mm, *d* = 1.5 mm and *S* = 4 mm, the response result is approximately equal to the pure-water value when *H*/*D* is greater than or equal to 0.30.

In the condition ofthe MCP with *D* = 20 mm, *h* = 2 mm, *d* = 1.3 mm and *S* = 4 mm, the response result is approximately equal to the pure-water value when *H*/*D* is greater than or equal to 0.25. In the condition of the MCP with *D* = 20 mm, *h* = 1 mm, *d* = 1.3 mm and *S* = 3 mm, the response result is approximately equal to the pure-water value when *H*/*D* is greater than or equal to 0.2. In Figure 4d, Figure 7d, Figure 10d and Figure 14, it is found that the MCP has good measurement characteristics for oil-water segregated flow in case of high *y_m_* or *H*/*D* in simulated calculation. Meanwhile, the MCP response result tends to be constant, i.e., pure-water correction for water content measurement, under the condition of *H*/*D* ∈ ((*h* + 3)/20, 1) or *y_m_* ∈ ((*h* + 2)/20, 1), which shows that the smaller the parameter *h* of the MCP is, the larger the low sensitivity sensitive area along the *Y*-axis in the sensor measurement field is, the smaller the critical value of *y_m_* or *H*/*D* which is needed for getting the pure water correction, and the easier obtaining the pure-water phase conductivity is.

## 4. Static Experiment for Oil-Water Segregated Flow

In order to avoid the polarization phenomenon, an alternating voltage source is adopted as the exciting signal in actual application, and the two electrodes are connected with a sinusoidal 20 kHz exciting signal in the measurement system. Meanwhile, in order to avoid the voltage loss on the distance line, the V/F conversion circuit is adopted to construct the pure-water frequency measurement system.The instrument output is expressed by the frequency signal *F_m_* which can be described as:

(9) $$F_{m}=k_{V}\times V_{m}$$

where *k_V_* is correction coefficient, *V_m_* is the measuring voltage for pure water, pure oil or water-oil mixture, respectively.

**Figure 15:**
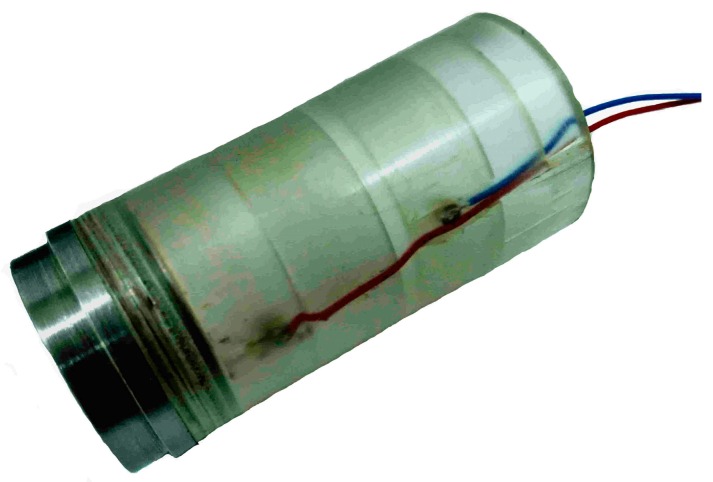
Images of the MCP with *D* = 20 mm and *L* = 50 mm.

**Figure 16:**
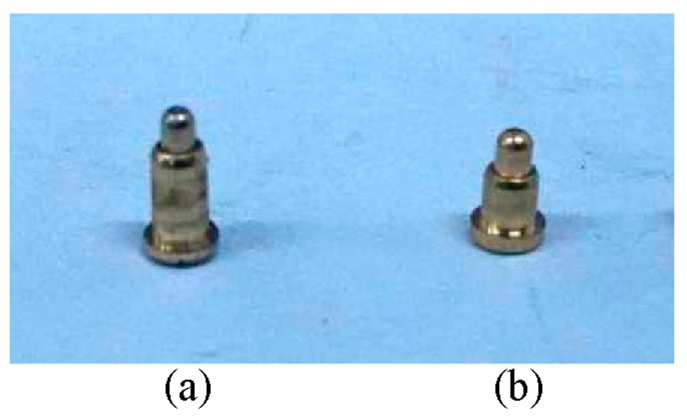
Images of the gold-plated electrodes: (**a**) *h* = 3 mm, *d* = 1.5 mm; (**b**) *h* = 2 mm, *d* = 1.3 mm.

Figure 15 shows images of the MCP. In Figure 15, it can be seen that the structures of the MCP are axially separated and flush-mounted on the inside wall of the insulated flow pipe with *D* = 20 mm. Figure 16 shows images of the gold-plated electrodes with different parameters. In Figure 16a, the gold-plated electrode *h* is equal to 3 mm and *d* is equal to 1.5 mm. In Figure 16b, the gold-plated electrode *h* is equal to 2 mm and *d* is equal to 1.3 mm.

To calibrate the MCP in the water-layer thickness measurement, the calibration test for oil-water segregated flow is carried out. The experimental mediums are tap water and industrial white oil. In the experiment, the fluid is forced into the insulated flow pipe of the MCP with *D* = 20 mm from a large diameter horizontal plexiglass pipe (the value of inner diameter is 50 mm). The different values of *y_m_* are matched in horizontal plexiglass pipe, and the range of oil holdup *y*_0_ is from 0% to 100% which is corresponding to the different values of *H*/*D*.

Considering that the measurement results of the instruments are influenced by the repeatability errors of the measurements and system errors during long time continuous operation, this paper employed horizontal oil-water two-phase flow experimental equipment to carry out the multiple (three or more times)repeated oil-water segregated experiments using an experimental point where *y_m_* and *H*/D change from 0 to 1, increasing 0.05 every time, whose result is recorded every 5 minutes and each experimental point is recorded 10 times.

**Figure 17:**
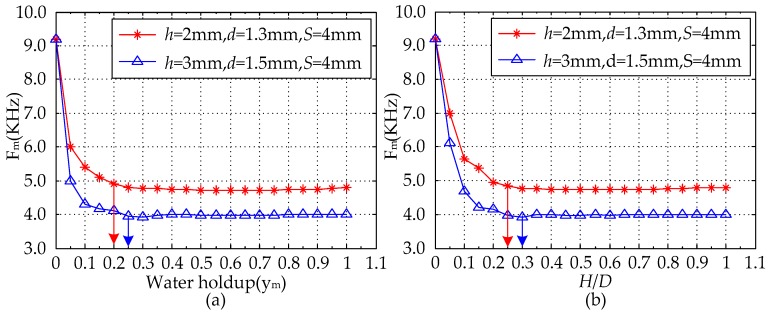
Calibration of the MCP with two different structure parameters in the condition of oil-water segregated distribution (*D* = 20 mm, *L* = 50 mm): (**a**) *F_m_* vs. *y_m_*; (**b**) *F_m_* vs. *H*/*D*.

Figure 17 shows the comparison of the MCP with different structure parameters measuring the horizontal oil-water segregated distribution response results. As can be observed from Figure 17, the range of both *y_m_* and *H*/*D* are from 0 to 1, which can be described as: when *y_m_* = 0 or *H*/*D* = 0, the measured result of the MCP is the pure-oil value; when *y_m_* = 1 or *H*/*D* = 1, the measured result of the MCP is pure-water value; and when 0 < *y_m_* < 1 or 0 < *H*/*D* < 1, the measured result of the MCP is between the pure-oil value and the pure-water value. The measured values of the MCP with different structure parameters in the conditions of pure oil and pure water are shown in Table 3. As can be seen, the measured MCP values have obvious differences for the conditions of pure oil and pure water, which can be attributed to the distinctive resistivity differences of water and oil. Meanwhile, when *y_m_* = 0 or *H*/*D* = 0, i.e., the condition of pure oil, the measured values of sensors with different structure parameters are exactly the same, which can be attributed to the fact that the measured voltage reaches saturation in the actual circuit system. When *y_m_* = 1 or *H*/*D* = 1, i.e., the condition of pure water, the measured values of the sensors with different structure parameters are different, which can be attributed to the different electric field or high sensitivity sensitive area generated by the two electrodes in the measurement field. With the increase of *y_m_* or *H*/*D*, under the conditions of lower *y_m_* or *H*/*D*, the measured frequency signal *F_m_* of the MCP with two different structure parameters shows obvious differences and decreasing tendency, which could be attributed to the stronger electric field in the measurement region, where the non-conducting oil phase is located in the high sensitivity sensitive area analyzed from Figure 4, Figure 7 and Figure 10. When *y_m_* or *H*/*D* is greater than or equal to a constant value, the response result of the MCP is pretty close to the pure-water value, and it is gently influenced by the non-conducting oil phase, which can be attributed to the non-conducting oil phase being away from the high sensitivity sensitive area. Therefore, under the conditions of higher *y_m_* or *H*/*D*, the MCP can obtain a reliable and accurate pure-water value, which can be used as the pure-water correction for water content measurements in horizontal oil-water segregated flow. As can be seen from Figure 17a, in the condition of the MCP with *D* = 20 mm, *h* = 3 mm, *d* = 1.5 mm and *S* = 4 mm, the response result *F_m_* is pretty close to pure-water value when *y_m_* is greater than or equal to 0.25. In the condition of the MCP with *D* = 20 mm, *h* = 2 mm, *d* = 1.3 mm and *S* = 4 mm, the response result *F_m_* is pretty close to pure-water value when *y_m_* is greater than or equal to 0.20. As can be seen from Figure 17b, in the condition ofthe MCP with *D* = 20 mm, *h* = 3 mm, *d* = 1.5 mm and *S* = 4 mm, the response result *F_m_* is pretty close to the pure-water value when *H*/*D* is greater than or equal to 0.30. In the condition of the MCP with *D* = 20 mm, *h* = 2 mm, *d* = 1.3 mm and *S* = 4 mm, the response result *F_m_* is pretty close to pure-water value when *H*/*D* is greater than or equal to 0.25.

**Figure 18:**
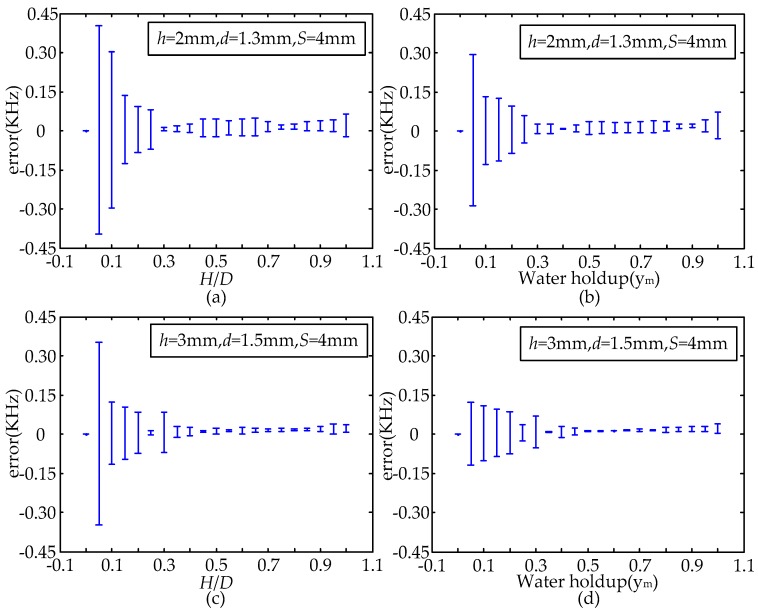
Error bars of the MCP with different structure parameters from two aspects of *y_m_* and *H*/*D* (*D* = 20 mm, *L* = 50 mm): (**a**,**b**) *h* = 2 mm, *d* = 1.3 mm, *S* = 4 mm; (**c**,**d**) *h* = 3 mm, *d* = 1.5 mm, *S* = 4 mm.

Considering the influence of the repeatability errors of measurement and system errors comprehensively, the measurement error of the experimental point can be obtained by calculating the mean square error for the data set of the repeated experiments and long time records to the corresponding experimental point. The error bars of the MCP with two different structure parameters in the conditions of *y_m_* and *H*/*D* are shown in Figure 18. As can be seen from Figure 18, the measured error of each structure sensor has obvious difference in the conditions of *y_m_* or *H*/*D*, under the condition of lower *y_m_* or *H*/*D*, the distribution of measured error has a great fluctuation, which can be attributed to the weak fluctuation of the fluid in the measurement region, where the non-conducting oil phase is located in the high sensitivity sensitive area. When *y_m_* or *H*/*D* is greater than or equal to a constant value, the measured error of the MCP is very small, which can be attributed to the fact that the non-conducting oil phaseis away from the high sensitivity sensitive area, and it indicates that the MCP can obtain an accurate pure-water value in horizontal oil-water segregated flow.

In Figure 4d, Figure 7d, Figure 10d and Figure 17, it is found that the MCP with *D* = 20 mm has good measurement characteristics for oil-water segregated flow in case of high *y_m_* or *H*/*D* in static experiments. Meanwhile, the MCP measured result tends to be constant, i.e., the pure-water value, in the conditions of *H*/*D* ∈ ((*h* + 3)/20, 1) or *y_m_* ∈ ((*h* + 2)/20, 1), which shows that the smaller the parameter *h* of the MCP is, the larger the low or non-sensitive area along the *Y*-axis in the sensor measurement field is, the smaller the critical value of *y_m_* or *H*/*D* which is needed for getting pure-water correction, and the easier obtaining pure-water phase conductivity is.

From the comprehensive analysis of the simulation calculation and static experiment, under the condition of *H*/*D* ∈ ((*h* + 3)/20, 1) or *y_m_* ∈ ((*h* + 2)/20, 1), the measured value of the MCP in the horizontal oil-water segregated flow is approximately equal to the pure-water value, the MCP has good characteristics such as being stable and reliable for pure-water correction measurements with the continuous water phase, and the sensors whose value of the parameter *h* is minor have great adaptability in horizontal oil-water segregated flow.

Therefore, the response result of the MCP could be given as the calibration result of pure-water phase conductivity works satisfactorily in static experiments of oil-water segregated flow. Furthermore, the critical value of *y_m_* or *H*/*D* which is needed for getting the pure-water correction is not only related to the parameter *h*, but also the parameter *D*, simultaneously. Therefore, it is necessary to further investigate the effect of the parameter *D* on the performance of MCP in the future. Also the flow loop test will be carried out in the future to further examine the performance of the MCP.

## 5. Conclusions

In this work, a novel sensor is presented in which the MCP is used in horizontal oil-water segregated flow. The simulation model of this structure of the MCP is established in the FEM. Simulation experiments of electric field distribution and the YoZ plane element sensitivity distribution of the MCP measurement area were conducted. The optimization indexes, i.e., the parameters *S_avg_* and *S_vp_*, are adopted to study the effect of sensor geometry on the sensitivity field, and to obtain appropriately characteristic parameters through the analysis of two performance indicators, i.e., electric field and element sensitivity of the MCP. The parameters *S* and *d* when the parameter *h* has different values after optimization are the following: when *h* = 3 mm, *S* = 4 mm and *d* = 1.5 mm; when *h* = 2 mm, *S* = 4 mm and *d* = 1.3 mm; and when *h* = 1 mm, S = 3 mm and *d* = 1.3 mm. Finally, combining the FEM simulation calculation and static experiments on oil-water segregated flow, the measurement characteristics of the MCP are analyzed from two aspects of *y_m_* and *H*/*D*, which indicates that the response result of the MCP could be given as the pure-water calibration result in the conditions of *H*/*D* ∈ ((*h* + 3)/20, 1) or *y_m_* ∈ ((*h* + 2)/20, 1), and the sensors whose value of the parameter *h* is minor have great adaptability in horizontal oil-water segregated flow. Meanwhile, the validity and feasibility of pure-water phase conductivity measurement with segregated flow in horizontal pipes are also verified by experiment results.

## Figures and Tables

**Table 1:** Sensitivity field parameters of MCP with different grids (*D* = 20 mm, *h* = 3 mm, *d* = 1.5 mm, *S* = 4 mm).

No.	Total Grids	Maximum Grid/m^3^	Minimum Grid/m^3^	Uo (V)	*S_avg_*	*S_dev_*	*S_vp_*
1	24,275	7.33 × 10^−5^	1.46 × 10^−6^	1.273	0.105	0.185	1.768
2	39,315	5.17 × 10^−5^	1.41 × 10^−6^	1.275	0.101	0.186	1.850
3	57,601	6.22 × 10^−5^	1.42 × 10^−6^	1.276	0.104	0.186	1.795
4	74,299	3.85 × 10^−5^	1.41 × 10^−6^	1.276	0.103	0.188	1.810
5	101,082	3.97 × 10^−5^	1.20 × 10^−6^	1.276	0.102	0.189	1.848

**Table 2:** The calculated response of the MCP with different structure parameters in the conditions of pure oil and pure water.

*D* = 20 mm	*V_m_* vs. *y_m_* or *H*/*D* (V)	
	Pure Water (*y_m_* = 1 or *H*/*D* = 1)	Pure Oil (*y_m_* = 0 or *H*/*D* = 0)
*h* = 3 mm, *d* = 1.5 mm, *S* = 4 mm	1.276	1.28 × 10^11^
*h* = 2 mm, *d* = 1.3 mm, *S* = 4 mm	1.867	1.87 × 10^11^
*h* = 1 mm, *d* = 1.3 mm, *S* = 3 mm	2.514	2.51 × 10^11^

**Table 3:** The measured values of the MCP with different structure parameters in the conditions of pure oil and pure water.

*D* = 20 mm	*F_m_* vs. *y_m_* or *H*/*D* (KHz)	
	Pure Water (*y_m_* = 1 or *H*/*D* = 1)	Pure Oil (*y_m_* = 0 or *H*/*D* = 0)
*h* = 3 mm, *d* = 1.5 mm, *S* = 4 mm	4.00	9.20
*h* = 2 mm, *d* = 1.3 mm, *S* = 4 mm	4.80	9.20
